# Concurrent and aerobic exercise training promote similar benefits in body composition and metabolic profiles in obese adolescents

**DOI:** 10.1186/s12944-015-0152-9

**Published:** 2015-11-26

**Authors:** Paula Alves Monteiro, Kong Y. Chen, Fabio Santos Lira, Bruna Thamyres Cicotti Saraiva, Barbara Moura Mello Antunes, Eduardo Zapaterra Campos, Ismael Forte Freitas

**Affiliations:** Center and Prescription Motor Activity Laboratory, Department of Physical Education, Universidade Estadual Paulista, UNESP, Rua Roberto Simonsen, 305, 19060-900 Presidente Prudente, São Paulo Brazil; Exercise and Immunometabolism Research Group, Department of Physical Education, Universidade Estadual Paulista, Presidente Prudente, São Paulo Brazil; Diabetes, Endocrinology, and Obesity Branch, National Institute of Diabetes and Digestive and Kidney Disease, National Institutes of Health, Bethesda, MD USA

**Keywords:** Body fat, Body composition, Exercise, Lipid profile, Obesity

## Abstract

**Background:**

The prevalence of obesity in pediatric population is increasing at an accelerated rate in many countries, and has become a major public health concern. Physical activity, particularly exercise training, remains to be a cornerstone of pediatric obesity interventions. The purpose of our current randomized intervention trial was to compare the effects of two types of training matched for training volume, aerobic and concurrent, on body composition and metabolic profile in obese adolescents. Thus the aim of the study was compare the effects of two types of training matched for training volume, aerobic and concurrent, on body composition and metabolic profile in obese adolescents.

**Methods:**

32 obese adolescents participated in two randomized training groups, concurrent or aerobic, for 20 weeks (50 mins x 3 per week, supervised), and were compared to a 16-subject control group. We measured the percentage body fat (%BF, primary outcome), fat-free mass, percentage of android fat by dual energy x-ray absorptiometry, and others metabolic profiles at baseline and after interventions, and compared them between groups using the Intent-to-treat design.

**Results:**

In 20 weeks, both exercise training groups significantly reduced %BF by 2.9-3.6 % as compare to no change in the control group (*p* = 0.042). There were also positive changes in lipid levels in exercise groups. No noticeable changes were found between aerobic and concurrent training groups.

**Conclusions:**

The benefits of exercise in reducing body fat and metabolic risk profiles can be achieved by performing either type of training in obese adolescents.

**Trial registration:**

Registration number: RBR-4HN597.

## Background

The prevalence of obesity in pediatric population is increasing at an accelerated rate in many countries, and has become a major public health concern [1]. Childhood obesity is one of the main predictor factors of adult obesity [2], with a high risk that an obese adolescent has 80 % chance of becoming an obese adult [3]. As a country with an emerging economy, data from Brazilian Institute of Geography and Statistics (IBGE) show that 4 % of female and 5.9 % of male adolescents are obese [4].

Obesity is characterized by excess body fat accumulation resulting from genetic and behavioral factors that lead to chronic positive energy balance [5]. Adipose tissue is metabolically active that contributes significantly to the overall homeostasis by secreting inflammatory cytokines [6]. Obesity is associated with chronic low-grade systemic inflammation that may have a causal role in the development of several diseases. This inflammation is directly related to excess body fat, especially visceral fat [7]. This inflammatory milieu has direct influence on high concentrations of lipid and glucose profiles and the insulin levels [8], thus impacting major organs such as the heart, liver, and kidneys.

Physical activity, particularly exercise training, remains to be a cornerstone of pediatric obesity interventions [9, 10]. Some main objectives of exercise for the treatment and prevention of obesity are: to reduce body fat, stabilize concentrations lipids, glycemic, and hormones such as insulin and cytokines produced by adipose tissue. In addition, an increase in fat-free body mass, mediated by physical exercise, may be beneficial to energy metabolism and the production of anti-inflammatory cytokines [11].

Aerobic exercise is a commonly recommended model for obese individuals, with beneficial effects on body composition, lipid profile, blood pressure, glycemic control and cardiorespiratory fitness [10]. In comparison, resistance training increases muscle mass, strength, and endurance, and increased bone mineral density [9]. Concurrent training, designed to activate two different metabolic pathways (aerobic and anaerobic) thus is thought to enhance the effects of both aerobic and resistance training models.

The purpose of our study was to compare the effects aerobic and concurrent on body composition and metabolic profile in obese adolescents in a randomized control trial. Our primary hypothesis was that the concurrent training would significantly decrease total percent fat (% fat) in comparison to the control group in 20 weeks. Our explorative hypotheses were that, standard aerobic training, if matched for training frequency and volume, would have comparable effects as compared to the concurrent training group, and that individual baseline metabolic profiles could contribute to the effectiveness of training.

## Methods

### Participant characteristics

This research was approved by the Research Ethics Committee of the *Universidade Estadual Paulista*, São Paulo, Brazil (protocol number 07/2009). Recruitment of obese adolescents to participate was advertised by local press (newspaper, television and internet). Potential volunteers, who identify themselves as obese, contacted the group staff and were invited to attend in the Laboratory at the University, with their parents or legal guardians for the initial screening. After explaining the study, the adolescents who fit the inclusion criteria signed consent documents by his/her parent or legal guardian, including an assent from the child. Adolescents who participated in the control group were included in the inclusion criteria and underwent the same evaluations of adolescents who trained, however for logistic reasons could not attend intervention.

The inclusion criteria were: (a) between 11 and 17 years of age; (b) considered to be obese of according to BMI, adjusted for age and gender, outlined by Cole et al. [12]. Anyone with major diseases or clinical indications that prevents physical activity was excluded. During screening, each participant was also submitted to a fasted blood sample, abdominal ultrasound, maximum stress test, anthropometry, and body composition by DEXA. All evaluations were repeated in the identical condition at after 20 weeks.

### Study design

This was a parallel design to study the impact of two types of exercise training in obese adolescents as compare to a control group with no intervention. None of the group received dietary interventions. The two exercise groups were randomized and the data analyses were performed by a blinded investigator (KYC). The study duration was 20 weeks, with all the adolescents being evaluated at the baseline and at the end of the study (between 3 to 5 days of the last training session).

### Assessment and tests

#### Anthropometry

Body mass was measured in an electronic scale (Filizzola PL 150, Filizzola® Ltda, Brazil). Body height and the trunk-head length were measured with a wall-mounted stadiometer (Sanny®, São Paulo, Brazil). The length of lower limb was calculated by the difference between height and trunk-head height. Peak height velocity (PHV) [13] and BMI were determined.

Waist circumference (WC) was measured at lowest circumference between the superior border of the iliac crest and below the lowest rib with a inelastic tape (Sanny®, São Paulo, Brazil), with the subjects in a standing position and breathing normally and with arms relaxed beside the trunk. The record was made at the end of a normal expiration. All anthropometric measurements were made following the recommendations proposed by Lohman et al. [14].

#### Body composition

Body composition and fat distribution was measured by a DEXA scanner (Lunar DPX-NT, General Electric Healthcare, Little Chalfont, Buckinghamshire, United Kingdom, software version 4.7). The outcome variables were percentage body fat (%BF), percentage fat android (%FA), fat-free mass (FFM) (kg), and total fat mass (FM) (kg). All measurements were made in a climate-controlled temperature room and according to the standard operating procedures recommended by the manufacturer. The reproducibility of total body %BF, FM, and FFM using DEXA has been reported to range from 0.8-1.6 % coefficient of variation (CV) in adults [15, 16]. Our internal data showed a test-retest CV of 1.48 % for % BF measurements in *n* = 12 post-menopausal women [17].

#### Ultrasound

The intra-abdominal adiposity tissue (IAAT) (cm), subcutaneous adiposity tissue (SAT) (cm), and hepatic steatosis were measured by an ultrasound examination of the upper abdomen. SAT was measured from the distance between the outer skin and abdominal rectus muscle, IAAT was defined as the distance between the inner wall of the abdominal cavity and the anterior wall of aorta [18]. The hepatic steatosis diagnostic criteria followed the following specifications: (i) the absence of normal echogenicity and (ii) the presence of change of fine echoes, diaphragm viewing and borders of the blood vessels intra hepatic [19]. All adolescents followed the recommendation to fast for at least 4 h prior to the evaluation. And the examination was performed by only one qualified radiologist, using a TOSHIBA Eccocee scanner with a 3.7 Mhz convex transducer (Tokyo, Japan).

#### Blood samples

Fasting blood samples (20 ml) were taken into two vacutainer tubes containing EDTA (5 ml each) for plasma separation and two dry vacutainer tubes (5 ml each) for serum separation. After collection, tubes was centrifuged at 3.000 RPM for 12 min at 4 °C, and plasma and serum samples were stored at −20 °C until analyses. The blood samples were performed by registered nurses and the biochemical analyses were done in a Laboratory of the University. Participants were instructed to refrain from performing physical exercises (exercise or unusual activities) for 72 h and to maintain a 12 h fasting before the test.

#### Blood analyses

Total cholesterol (TC), triacylglycerol (TG), high density lipoprotein (HDL), low density lipoprotein (LDL), very low density lipoprotein (VLDL) and glucose, were assessed using commercial kits (Labtest®, São Paulo, Brazil). Non-ester fatty acid (NEFA) was assessed by a colorimetric method with a commercial kit (Wako Diagnostics, Mountain View, CA 94043 USA). Cytokines (IL-6, IL-10 and TNF-α) were assessed using Enzyme-Linked Immunosorbent Assay (ELISA) commercial kits (affimetrix/eBioscience, Ambriex S/A, São Paulo, Brazil) and the PAI-1 was measured using kit of assay R&D Systems (R&D Systems, Abingdon, UK).

#### Maximum stress test

The participants performed maximal exercise testing on a treadmill (Inbrasport ATL 2000, maximum capacity of180 kg, 0-26 % incline and speed of up to 24 km / h). The adolescents were instructed not to perform physical exercise during 24 h prior and remain fasted for at least 3 h before the test. All participants were evaluated in the afternoon. Before the test the adolescents conducted a warm up of 5 min at a velocity of 5 km/h. The test started at a velocity of 3.5 km/h and an inclination of 1 %. Every minute there was increment 0.5 km/h in the velocity [20]. During the entire test the participants’ heart rate was continuously monitored, and the blood pressure was measured. Through the stress test, the VO_2peak_ and peak heart rate were established and used to design the individual intensities of aerobic training.

#### Prediction test of a maximum repetition

The adolescents performed the 10 maximum repetition test (10RM) [21]. Leg press (45° angle), bench press (supine), low rowing (sitting with no back support) and biceps curl (standing) were used to assess the strength of upper and lower body large muscle groups (Ipiranga, Presidente Prudente, Brazil).

### Interventions

#### Control

The adolescents were instructed not to change their usual eating or physical activity behavior during the entire 20 weeks. To enhance compliance of the control participants, they were included for future obesity intervention studies.

#### Aerobic training

Aerobic training was performed three times per week, with each session consisted of 50 min and consisted in walking and running. At the beginning two weeks of the intervention, the adolescents began at Stage 1 which consisted of exercising at a moderate intensity (13 and 14 of the Borg scale, with a full scale of 6–20), with the intended training intensity between 65 % and 85 % of VO_2peak_. The intensity of the effort was monitored by a heart rate monitor (Polar® S810, Finland) and progressively increased through Stage 6 such that the participants remained in the aerobic training zones in the protocol (Table 1).

**Table 1:** Intensity of aerobic training

	N° of weeks	Intensity
Stage 1	2	13 to 14 of Borg scale
Stage 2	2	65 % of VO_2 peak_
Stage 3	4	70 % of VO_2 peak_
Stage 4	4	75 % of VO_2 peak_
Stage 5	4	80 % of VO_2 peak_
Stage 6	4	85 % of VO_2 peak_

#### Concurrent training

The concurrent training was performed at three times per week, with each session consisted of 60 min of 50 % of resistance training time followed by 50 % of the aerobic training (identical to the aerobic training protocol but only for 30 min) [22].

Similar to the aerobic training group, the resistance exercise began at Stage 1 with minimal loads at each session, and increased to an intensity of 55 % of RM for the next two weeks. The intensity was progressively increased every four weeks with the final intensity of 75 % of RM (Table 2). The resistance exercise routine was conducted in the form of circuit training in the following order: leg press, low rowing, bench press, squat rack, seated lat pull-down, leg curl, arm curl, seated chest fly, triceps, leg extension, sit up, and supine trunk extension.

**Table 2:** The resistance training intensity

	N° of weeks	Intensity	N° of sets	Repetitions/set
Stage 1	2	Minimum loads	1	20
Stage 2	2	55 % RM	1	20
Stage 3	4	60 % RM	1	20
Stage 4	4	65 % RM	1	20
Stage 5	2	70 % RM	2	10
	2		2	12
Stage 6	2	75 % RM	2	12
	2		2	15

#### Matching workload between concurrent and aerobic training

In order to have comparable total workloads for the concurrent and aerobic training groups, we designed the exercise protocols by the training impulse calculation method (TRIMP), which is a method of assessing the volume and the intensity of each session by specific scores at any stage [23].

To match the total amount of workload during each session between the aerobic and concurrent groups, we performed pilot trials in attempt to match the sums of heart rate over the entire exercise periods (50 min of aerobic vs. 30 min of aerobic + 30 min of resistance exercises) and adjusted the intensities and durations accordingly.

### Data analyses and statistics

Descriptive data are shown as means and standard deviation. We performed a power analysis for the design of this study, and based on the observation from our previous study in obese adolescents that a 16-week of concurrent training resulted in a reduction of % BF by 3.4 % ± 2.8 % [24]. Our primary hypothesis was that the reduction in total %BF in the concurrent training group of obese adolescents after 20 weeks of training would be statistically significant as compared to the control group assessed at the same time period, with a power (1-type II error) of 0.80 and a type I error of 0.05 (two-tailed design using the independent *t*-test). Using PS software (ver 3.1.2, Dupont and Plummer, http://biostat.mc.vanderbilt.edu/wiki/Main/PowerSampleSize), it was estimated that we would need 12 subjects per group. Considering a dropout rate of 25-40 %, we over-recruited the number of subjects. We used the intend-to-treat (ITT) analysis with all subjects in the three groups (concurrent, aerobic, and control) after randomization regardless if they completed the training. In addition, we want to compare the effects of the concurrent training to standard aerobic training. One-way analysis of variance (ANOVA) with Tukey’s post hoc analysis was applied to compare the 20-week changes in body composition, lipids and inflammatory as the primary analysis. For the explorative analyses, 20-week changes in body composition and metabolic profiles were associated with gender, growth maturation, training type, and hepatic steatosis by multiple regression analysis. The statistical analyses were conducted using SPSS, version 17.0 (SPSS Inc. Chicago. IL).

## Results

**Fig. 1:**
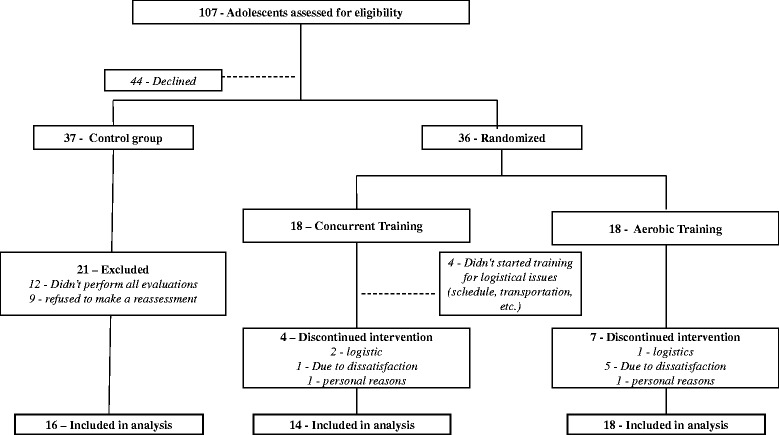
Control, aerobic and concurrent training groups in trial flow diagram

We conducted two waves of recruitments in October 2013 and March 2014. After initial screening, qualified participants were assigned to three different groups (control, concurrent training, and aerobic training, with the exercise groups being randomized). The randomization was stratified according to sex and age. As shown in Fig. 1, 107 adolescents attended the initial screening, and 73 qualified and agreed to participate. In the control group, 16 adolescents completed both baseline and follow-up measurements. In the concurrent group, four adolescents did not begin the training sessions due to logistics reasons. Thus, the final analyses were performed on 48 adolescents (16 controls, 14 concurrent, and 18 aerobic, respectively).

At baseline, age, %BF, and lipids parameters were similar between all three groups. In the two exercise groups, 11 adolescents from the aerobic training and 10 from the concurrent training successfully completed all the designed intervention sessions, respectively. However, ITT analyses were performed on the entire study cohort.

**Fig. 2:**
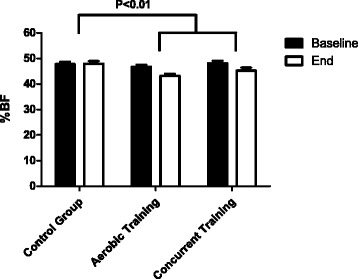
Effect of training in the percentage of body fat after 20 weeks of intervention in obese adolescents

As our primary outcome set *a priori*, both exercise training groups significantly changed %BF as compared to the control group (*p* = 0.042). More specifically, in the 20 weeks, while %BF in the control group slightly did not change (0.16 ± 1.67 %, p = 0.71), the concurrent and the aerobic training resulted in significant decreases in %BF (−2.85 ± 3.05 %, *p* = 0.004; and −3.59 ± 2.32 % (*p* = 0.0001), respectively). Interestingly, there was no difference between the two training groups (Fig. 2).

The specific data was shown in Table 3. The changes in % AF (*p* = 0.001), IAAT (*p* = 0.035), TG (*p* = 0.000), HDL (*p* = 0.030) and VLDL (*p* = 0.000) were also different between groups.

**Table 3:** Baseline and end measurements in obese adolescents, expressed in mean (standard deviation)

	Control (*n* = 16)		Aerobic Training (*n* = 18)		Concurrent Training (*n* = 14)	
	Baseline	End	Baseline	End	Baseline	End
Age (years)	11.04(1.90)	11.32(1.90)	11.00(1.02)	11.42(1.02)	11.03(1.34)	11.42(1.34)
Body mass (kg)	77.60(16.97)	80.20(17.09)^b^	74.08(12.13)	73.02(11.43)^a^	86.67(12.98)	86.46(11.32)^a^
BMI (kg/m^2^)	30.95(3.42)	32.04(34.48)^a^	30.15(2.90)	28.71(3.02)^ab^	33.17(4.7)	32.36(4.46)^a^
Sex	8 girls 8 boys		8 girls 10 boys		5 girls 9 boys	
Body Composition						
*%BF*	47.77(3.57)	47.98(3.94)^b^	46.71(3.28)	43.12(3.74)^a^	48.06(3.89)	45.21(4.53)^a^
*FM (kg)*	36.94(7.44)	38.16(6.80)	34.73(7.04)	31.61(6.50)	42.02(9.76)	39.41(8.74)
*FFM(kg)*	37.89(9.33)	39.59(10.98)	36.97(5.58)	38.83(5.66)	42.07(4.04)	44.42(3.91)
*%AF*	55.58(2.80)	55.96(2.56)^b^	53.82(4.14)	49.83(4.41)^a^	56.07(2.78)	52.56(3.32)^a^
*Waist (cm)*	93(9.33)	95.52(11.80)^b^	89.77(5.81)	85.52(6.08)^a^	97.29(10.65)	93.52(7.55)^b^
*IAAT(cm)*	3.57(1.13)	3.58(1.06)^b^	3.35(0.98)	2.53(0.99)^a^	4.11(1.70)	4.05(1.67)^ab^
*SAT(cm)*	3.15(0.63)	2.96(0.58)	2.72(0.64)	2.58(0.72)	2.91(0.97)	3.04(0.62)
Lipids						
*CHO (*mg/dl*)*	150.60(28.63)	130.19(33.14)	142.12(28.42)	112.52(25.20)	150.64(54.97)	110.38(18.85)
*TG (*mg/dl)	134.86(55.88)	154.30(46.41)^b^	153.13(28.07)	121.21(34.86)^a^	144.02(36.40)	117.74(26.10)^a^
*HDL (*mg/dl)	42.86(8.67)	36.33(11.88)^b^	34.85(3.70)	37.91(10.21)^ab^	36.90(3.06)	42.39(12.24)^a^
*LDL (*mg/dl)	79.46(23.95)	69.06(27.57)	76.42(29.31)	50.36(27.79)	74.51(18.40)	40.60(20.77)
*VLDL (*mg/dl)	28.26(12.99)	30.86(9.28)^b^	30.62(5.62)	24.24(6.97)^a^	28.80(7.28)	23.54(5.22)^a^
*NEFA (*mmol/L)	0.97(013)	1.00(0.105)	1.01(0.12)	0.93(0.075)	1.01(0.067)	0.94(0.094)

*%BF* Percentage of body fat*, FFM* Fat free mass*, %AF* Percentage android fat*, IAAT* Intra-abdominal adiposity tissue*, SAT* Subcutaneous adiposity tissue*, CHO* Total cholesterol, very low density lipoprotein*, TG* triacylglycerol, *HDL* high density lipoprotein, *LDL* low density lipoprotein, *VLDL* very low density lipoprotein*;. Lowercase letters difference between groups p <0.05 by ANOVA (ITT analyses). a, b means that the changes between baseline and end measures were different from each other, and ab means that it is similar to either a and b*

To explore what parameters can impact the individual changes in % BF and other metabolic profile parameters (dependent variables),  we included gender, age, BMI, PHV (a continuous measure of maturation), the presence or absence (1/0) of hepatic steatosis, IAAT at baseline, and the type of training performed as potential independent factors. Our multiple regression analysis revealed that only the type of training performed independently predicted the changes in % BF after 20 weeks. Moreover, baseline hepatic steatosis and IAAT were independent predictors of the changes in hepatic steatosis, and the type of training and baseline VLDL independently predicted the changes in VLDL (Table 4).

**Table 4:** Summary of independent associations between training types, baseline characteristics and the changes of body composition and metabolic profiles by multiple linear regression analysis.

	Unstandardized coefficients				Standardized Coefficients	
Changes (end-baseline)/predictors	B	Std. error	Adjusted R square	t	Beta	*p*-value
% Body Fat						
Type of training^1^	−1.825	0.442	0.267	−4.129	−0.53	<0.001
Constant	−0.180	0.597		−0.301		0.765
Hepatic steatosis^2^						
Baseline hepatic steatosis	−0.507	0.127	0.185	−3.999	−0.560	<0.001
Baseline IAAT^3^	0.107	0.046	0.265	2.336	0.327	0.025
Constant	−0.215	0.169		−1.274		0.210
VLDL^4^						
Type of training	−5.297	1.562	0.359	−3.391	−0.445	0.002
Baseline VLDL	−0.511	0.169	0.477	−3.028	−0.397	0.005
Constant	18.18	4.467		4.070		<0.001

^1^Type of training: 0= control group, 1= concurrent training, 2= aerobic training; ^2^Degree Hepatic steatosis: 0= ausence of hepatic steatosis, 1= presence of hepatic steatosis; ^3^Intra-abdominal adiposity tissue (IAAT) (cm); ^4^Very low density lipoproteins (VLDL) (mg/dl); ^5^Variables did not reach statistical significance: age, gender, baseline BMI, baseline % body fat, baseline growth velocity

## Discussion

Obesity is characterized as a worldwide epidemic that affects individuals of all ages and childhood and adolescence is a critical period for prevention and intervention efforts. Thus, the practice of regular exercise is seen as an important part in this process. There are divergences in the literature regarding various training models on reducing body fat in obese youth. Our study compared the effects of two types of exercise training modalities (aerobic and concurrent) for 20 weeks to a placebo control group in obese adolescents.

Our primary finding was that a significant decrease in total %BF was achieved in both training modalities as compared to the control group. The magnitude of %BF reduction in our study (−2.9 % and −3.6 % by concurrent and aerobic training) was comparable to Lee et al. (−2.6 % and −2.5 % by aerobic and resistance training groups) [25], but greater than the aerobic and resistance-only training groups in a recent study by Sigal et al. (−1.1 % to −1.6 %) [26]. Lee and colleagues only studied male adolescents, and the aerobic training frequency and intensities were similar to ours, and achieved the reduction in %BF in three months (12 weeks) while on a weight maintenance diet. The sample size (11–16 subjects per group) was also similar to ours, but the compliance of their study was around 99 % as compare to ours. Sigal et al. [26] had a much larger sample size (about 75 subjects per group, about 2:1 ratio of girls to boys) and the intervention was six months. Yet, despite of a prescribed −250 kcal/day energy deficit diet, only a moderate reduction in %BF (about −1 %) was observed in the aerobic group. Several possible factors could be contributing to this, such as that their exercise training was performed at gymnasiums perhaps with less personal monitoring as compared to ours.

The reductions in % AF (−3.5 % and −3.9 % by concurrent and aerobic training) were also significant (*p* = 0.001) when compared to the control group, suggesting that this effect could be beneficial to their metabolic profiles associated with abdominal obesity. Waist circumferences and visceral fat, as measured by abdominal ultrasound, decreased only in the aerobic training and was trending towards a decrease in concurrent training compared to the control group. The location of excess %BF is to be taken into consideration, because have a location more active metabolically and relation to chronic disease than total BF [27]. In this sense, as in other studies in the literature [25, 28], aerobic training shown to be more effective in treating and preventing chronic diseases associated with obesity, such as cardiovascular disorders and liver fat accumulation.

According Schranz N et al. [9], concurrent exercise promotes better results in body composition when compared to the resistance training and aerobic training alone. However, most previous studies did not match training volumes of concurrent versus other training modalities. Special efforts were made to our study design to carefully match the volume and the training intensity, which may have led to the similar training effects.

To our surprise, resistance exercises which were performed as part of the concurrent training in our study did not result in changes in FFM that increased, but not significantly, in all three groups of obese adolescents. In our control group, FFM increased as well as FM with time, which is likely due to normal growth and/or unhealthy weight gain. Both intervention groups gained FFM and lost fat mass. An increase in FFM in aerobic training group has been reported previously [25], likely due to weight bearing exercises help build muscle in addition to normal growth. We also know that resistance training can further promote an increase in FFM, and when combined with aerobic training, the benefits tend to be potentiated [29]. We did not find a group difference which could be due to small sample size. Interestingly, Sigal and colleagues [26] also did not detect any differences in total FFM amongst training groups and controls when combined with dietary restriction.

Compared to the control group, adolescents who underwent intervention with exercise, both had an overall improved lipid profile with decreased concentrations of triacylglycerol and VLDL and an increase in HDL (Table 3). Exercise training has been shown to decreased concentration of triacylglycerol plasma and VLDL in adults [30] and in adolescents [31]. Exercise increases HDL levels in adults [32] and in youth [33]. The contribution by the changes in total fat mass in our study is interesting, as the exercise types did not change the outcomes in HDL independently. This could be due a small sample size. Realizing that obesity is a chronic inflammatory state, we also explored the baseline and post-intervention levels of cytokines such as TNF-alpha, IL-6, IL-10, and PAI-1. As expected, there were not group differences neither at baseline nor the changes after intervention (data not shown). It has been shown that these markers have link in adults, and limited data in obese adolescents are available, especially the changes after interventions.

We also measured fasting serum glucose and insulin as exploratory outcomes. Because none of the adolescents were diabetic, the glucose levels did not differ at baseline between groups, and did not change significantly after 20 weeks (data not shown). It is possible that, because they are going through a transient insulin resistance stage [34], the levels of glucose and insulin can be highly variable, and our study did not have the power to detect any meaningful changes.

To separate the potential confounding variables such as sexual maturation, gender, and even the presence of some metabolic disorders to the effect of interventions on BF, we have shown (through multiple regression analysis) that the only independent factor capable of producing the observed changes in %BF and VLDL was exercise training. Interestingly, the presence of hepatic steatosis at baseline independently predicted the changes in IAAT, such that adolescents who had steatosis lost more intra-abdominal fat in both exercise groups. This preliminary evidence suggests that the beneficial effect of exercise in adolescents with hepatic steatosis may involve the changes in visceral fat, which has been implicated with worse metabolic conditions such as diabetes.

Overall, the advantages of our study were: [1] the controlled interventions performed at a designated center with trained staff led to significant fat loss in obese adolescents receiving both exercise training; [2] we matched training volume between aerobic and concurrent sessions; [3] we measured clinical parameters such as lipids, IAAT and steatosis together with our primary outcomes of body composition; [4] we further explored what contributed to the individual changes in %BF from potential confounders; and [5] we used a rigorous statistical analysis of Intent-to-treat.

However, the limitations were: [1] we had small number of adolescents in each arm, although due to the rigor of this study, such numbers were not uncommon and was statistically valid as designed; [2] we did not have a homogeneous population due to the location of our study; [3] we did not control energy intake during the study, which is well-recognized factor for adiposity development. However, we did not want to confound the potential impacts of exercise training on body composition by intervening dietary intake [4]. The control group was not randomized in the same fashion as the two exercise group due to the consideration of higher dropout rates in the control group, which despite of our efforts, we still incurred a dropout rate of 25-30 %, And [5] we did not directly measure the oxygen maximum consumption(VO_2max_) which would have been good to design individual adaptive exercise levels during the interventions.

## Conclusions

In summary, both the aerobic and concurrent training reduced total body fat percent in obese Brazilian adolescents as compared to the control group. These trainings also resulted in improvements of their lipid profiles (increased HDL and reduced triglycerides and VLDL). With matched training volume, the effects on %BF and FFM, and other metabolic parameters were similar between the two modes of exercise, suggesting that the benefits of exercise can be achieved by performing either type of training.
